# The role of warm ischemia time on functional outcomes after robotic partial nephrectomy: a radionuclide renal scan study from the clock randomized trial

**DOI:** 10.1007/s00345-023-04366-3

**Published:** 2023-04-21

**Authors:** Alessan dro Antonelli, Luca Cindolo, Marco Sandri, Alessandro Veccia, Filippo Annino, Francesco Bertagna, Fabrizio Di Maida, Antonio Celia, Carlo D’Orta, Bernardino De Concilio, Maria Furlan, Valentina Giommoni, Manuela Ingrosso, Andrea Mari, Roberto Nucciotti, Catia Olianti, Angelo Porreca, Giulia Primiceri, Luigi Schips, Francesco Sessa, Pierluigi Bove, Claudio Simeone, Andrea Minervini, The AGILE Group (Italian Group for Advanced Laparo-Endoscopic Surgery)

**Affiliations:** 1 Urology Unit, ASST Spedali Civili Hospital, University of Brescia, Brescia, Italy; 2 Urology Unit, Azienda Ospedaliera Universitaria Integrata Verona, AUOI Verona, University of Verona, 37126 Verona, Italy; 3 Urology Unit, D’Annunzio Hospital, University of Chieti, Chieti, Italy; 4 Big and Open Data Innovation Laboratory (BODaI-Lab), University of Brescia, Brescia, Italy; 5 Urology Unit San Donato Hospital, Arezzo, Italy; 6 Nuclear Medicine Unit ASST Spedali Civili Hospital, University of Brescia, Brescia, Italy; 7 Urology Unit, Careggi Hospital, University of Florence, Florence, Italy; 8 Urology Unit, San Bassiano Hospital, Bassano Del Grappa, Italy; 9 Urology Unit, Misericordia Hospital, Grosseto, Italy; 10 Nuclear Medicine Unit Careggi Hospital, University of Florence, Florence, Italy; 11 Urology Unit, Policlinico of Abano, Abano Terme, Italy; 12 Urology Unit, S. Carlo di Nancy Hospital, Rome, Italy

**Keywords:** Warm ischemia time, Robotic, Partial nephrectomy, On-clamp, Off-clamp, Renal tumor

## Abstract

**Purpose:**

To evaluate the relationship between warm ischemia time (WIT) duration and renal function after robot-assisted partial nephrectomy (RAPN).

**Methods:**

The CLOCK trial is a phase 3 randomized controlled trial comparing on- vs off-clamp RAPN. All patients underwent pre- and postoperative renal scintigraphy. Six-month absolute variation of eGFR (AV-GFR), rate of relative variation in eGFR over 25% (RV-GFR > 25), absolute variation of split renal function (SRF) at scintigraphy (AV-SRF).

The relationships WIT/outcomes were assessed by correlation graphs and then modeled by uni- and multivariable regression.

**Results:**

324 patients were included (206 on-clamp, 118 off-clamp RAPN). Correlation graphs showed a threshold on WIT equal to 10 min. The differences in outcome measures between cases with WIT < vs ≥ 10 min were: AV-GFR − 3.7 vs − 7.5 ml/min (*p* < 0.001); AV-SRF − 1% vs − 3.6% (*p* < 0.001); RV-GFR > 25 9.3% vs 17.8% (*p* = 0.008). Multivariable models found that AV-GFR was related to WIT ≥ 10 min (regression coefficient [RC] − 0.52, *p* = 0.019), age (RC − 0.35, p = 0.001) and baseline eGFR (RC − 0.30, p < 0.001); RV-GFR > 25 to WIT ≥ 10 min (odds ratio [OR] 1.11, *p* = 0.007) and acute kidney injury defined as > 50% increase in serum creatinine (OR 19.7, *p* = 0.009); AV-SRF to WIT ≥ 10 min (RC − 0.30, *p* = 0.018), baseline SRF (RC − 0.76, *p* < 0.001) and RENAL score (RC − 0.60. *p* = 0.028).

The main limitation was that the CLOCK trial was designed on a different endpoint and therefore the present analysis could be underpowered.

**Conclusions:**

Up to 10 min WIT had no consequences on functional outcomes. Above the 10-min threshold, a statistically significant, but clinically negligible impact was found.

**Supplementary Information:**

The online version contains supplementary material available at 10.1007/s00345-023-04366-3.

## Introduction

Partial nephrectomy aims at the complete cancer extirpation while saving as much healthy renal tissue as possible, given that population and institutional retrospective studies postulated a direct relationship between renal function and mortality [1–5]. In the recent years, the robot-assisted approach has been increasingly adopted, mainly due to its lower morbidity profile [6].

The evolution of renal function following RAPN is a multifactorial event relying on the quantity of preserved healthy renal parenchyma and its tolerance to the surgical insult. Among the multitude of patient’s, tumor’s, and surgical factors underlying these two domains [7], the clamping strategy drew most of the research efforts as the main modifiable surgical factor. Accordingly, since a few years ago, the urological community widely adopted the Thompson’s dogma claiming that “every minute counts” [8] so that many authors promoted strategies to avoid *at all* renal ischemia [9]. Further studies unveiled how ischemia time loses of significance when the quantity of spared renal tissue is accounted for, and the alternative concept that “every nephron counts” gained ground [5].

Thus, it is accepted that two kidneys patients with regular baseline function can well tolerate a “limited” warm ischemia time (WIT), but which is the optimal duration of such a limit remains a matter of debate. [10, 11]. The CLOCK (CLamp vs Off Clamp the Kidney during robotic partial nephrectomy) Trial is a multicenter prospective randomized trial whose primary endpoint was to compare the functional outcomes of on- vs off-clamp RAPN. All patients enrolled had split renal function measured by renal scintigraphy at baseline and 6 months after surgery. Herein we analyzed the CLOCK dataset to investigate whether the *duration* of WIT could influence postoperative global and split renal function.

## Materials and methods

### Study protocol of the CLOCK trial

The CLOCK trial (ClinicalTrials.gov NCT 02287987 [12]) is a phase 3 randomized controlled trial (RCT) conducted on behalf of the AGILE Group (Italian Group for Advanced Laparoscopic Surgery, http://www.agilegroup.it). Ethical committee approval was obtained by the coordinating center (NP 1814). Patients were consecutively recruited at 7 Italian Institutions between September 2015 and November 2018, with approximately constant accrual rate over time. One surgeon per institution with a well-defined profile (< 45 years-old, previous experience with at least 100 RAPN, done with both under on- and off-clamp approaches) performed all the procedures at his center. The data were collected within a web-based e-form maintained by independent data-managers; only the study statistician (M.S.) had access to the datasheet. The inclusion criteria were: age between 18 and 80 years-old; regular coagulation profile; baseline estimated glomerular filtration rate (eGFR) ≥ 60mL/min/1.73 m^2^; no abnormalities for both kidneys at medical history and imaging; cT1 renal tumor with RENAL score [13] complexity ≤ 10; agreement to participate to the trial and signed informed consent.

Pre- and postoperative management were handled by physicians/nurses not directly involved in the surgical procedures and blind of the randomization results. The CKD-EPI equation [14] was adopted to calculate eGFR from serum creatinine. Diethylene-triamine-pentacetic acid renal scan was prescribed before surgery and 6 months after to assess split renal function (SRF); nuclear medicine physicians were blinded of the surgical data. Postoperative acute kidney injury (AKI) was defined as increase > 50% in serum creatinine [15]. Follow-up included six-month abdominal and chest imaging and blood chemistry, up to 24 months from enrollment.

The surgical steps were strictly regulated by the study protocol: for both arms, kidney defatting and renal artery isolation were mandatory. In the on-clamp arm, at least the tumor resection and the inner renorrhaphy were required to be completed under global ischemia; in the off-clamp arm, the renal artery had to remain unclamped along all the procedure. Controlled hypotension during resection was not allowed. The specimens were examined according to the international guidelines by experienced uro-pathologists blinded from the clamping approach.

### Endpoints and statistical analysis of the present study

For the purposes of the present study, a single cohort was done by all the patients enrolled into the CLOCK trial, regardless their clamping approach. The aim of the study was to assess eventual relationships between WIT and the functional results of the procedure. The value of WIT was set as null (WIT = 0) for the off-clamp cases.

The functional outcomes were measured by the following endpoints assessed at 6 months after surgery:

- the absolute variation of eGFR (AV-GFR, continuous variable);
- the rate of relative variation in eGFR over 25% (RV-GFR > 25, dichotomous variable);
- the absolute variation in the SRF of the operated kidney at scintigraphy (AV-SRF, continuous variable).

The relationships between endpoints and the duration of WIT were here investigated by visual assessment, drawing scatter plots with a nonparametric regression line [16]. This preliminary analysis helped to identify potential nonlinear associations between the variables.

Uni- and multivariable regression were then used to model the relationships between WIT and response variables (linear regression for AV-GFR and AV-SRF by estimating regression coefficients [RC]; logistic regression for RV-GFR > 25 by estimating odds ratio [OR]). To account for potential confounders a set of variables defined *a priori* were entered into the models: age, gender, baseline eGFR, baseline SRF, RENAL score and occurrence of AKI.

This analysis was replicated also excluding the off-clamp cohort for which WIT was computed as null.

When scatter plots evidenced a nonlinear relationship, a piecewise linear regression function was used in the regression equation; the positions of knots were identified by visual assessment.

The distributions of continuous variables were summarized by median and interquartile range (IQR), while for categorical variables the number of observations with percentages (%) were reported. Differences between median values and proportions in subgroups of participants were tested using the Wilcoxon rank-sum test and Fisher’s exact test, respectively.

The statistical analyses were performed using Stata version 16.1 (Stata Corp., College Station, TX, USA). Statistical significance was set at *p* < 0.05.

## Results

This study dealt with a cohort of 324 patients, 206 undergone on-clamp, 118 off-clamp RAPN (Table 1—Supplementary Table 1). The baseline eGFR was 87.1 ml/min (IQR 75.9 to 96.5 ml/min) and the median SRF from the operated kidney was 49% (IQR 46–52%); the median duration of WIT was 10.5 min (IQR 0–16 min, range 0–34 min). At 6 months follow-up, the median AV-GFR was − 5.5 ml/min (IQR − 16 to 0 ml/min), the RV-GFR > 25 rate 14.8%, and the AV-SRF − 4.7% (IQR -12.8 to 0%).

**Table 1:** Full cohort baseline, perioperative, and pathological characteristics

Age (years)	65 (55–71)
Gender (male) *n* (%)	195 (60.2)
BMI (Kg/m^2^)	26.19 (24.34–28.57)
Charlson Comorbidity Index	0 (0–1)
Hypertension *n* (%)	180 (55.6)
Diabetes *n* (%)	38 (11.7)
Vasculopathy *n* (%)	53 (16.4)
Cardiopathy *n* (%)	69/322 (21.4)
Preoperative Hb (g/dL)	14.05 (13–15.14)
Creatinine at recruitment (mg/dL)	0.82 (0.71–0.95)
eGFR at recruitment (mL/min)	87.12 (75.91–96.52)
Split renal function operated kidney (%)	48.95 (46–51.73)
RENAL	6.5 (5–8)
Tumor dimension (cm)	3 (2–4)
Clamp *n* (%)	206 (63.6)
EBL (mL)	100 (50–150)
WIT (minutes)	10.5 (0–16)
OT (minutes)	120 (100–150)
LOS (days)	4 (3–5)
Postoperative complications n (%)	45 (14.2%)
Clavien grade *n* (%)	
1	20/45 (44.4)
2	15/45 (33.3)
3a	7/45 (15.6)
3b	2/45 (4.5)
4a	1/45 (2.2)
pT *n* (%)	
1a	234 (74.5)
1b	80 (25.5)
Malignant histology n (%)	241/315 (76.5)

*BMI* body mass index, *eGFR* estimated glomerular filtration rate, *EBL* estimated blood loss, *WIT* warm ischemia time, *LOS* length of stay

**Fig. 1:**
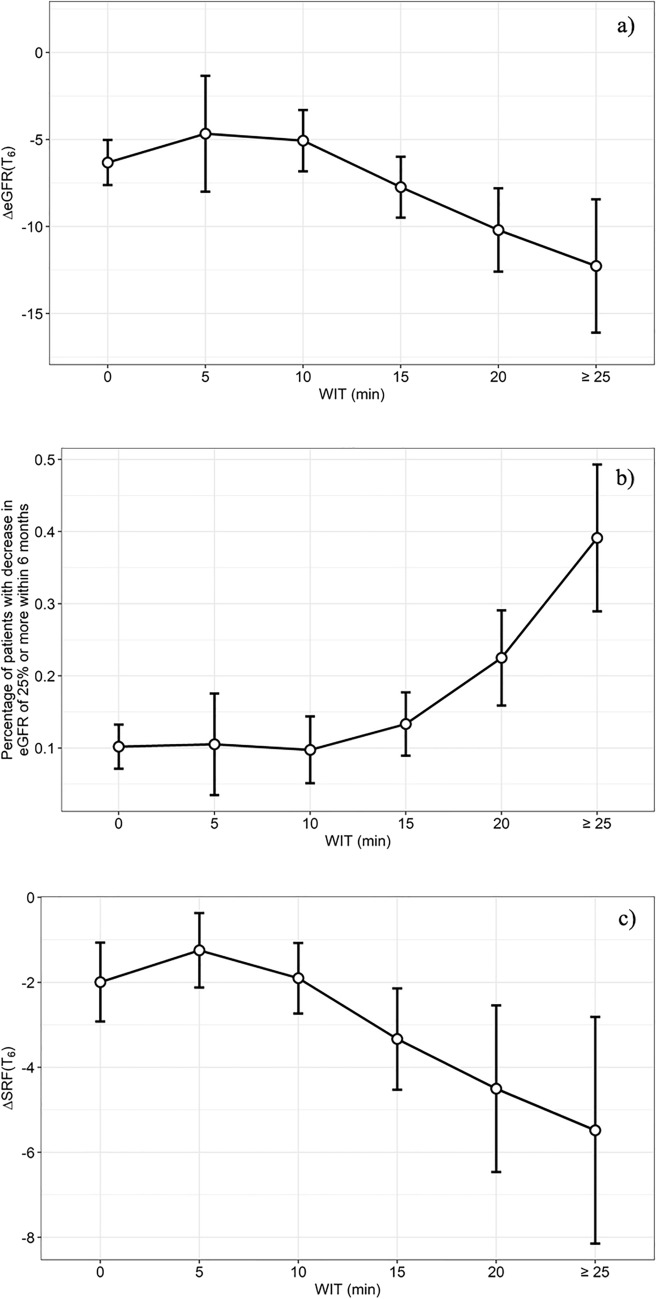
Visual assessment drawing scatter plots with a nonparametric regression line of **a** the absolute variation of eGFR (AV-GFR) at 6 months; **b** the rate of relative variation in eGFR over 25% (RV-GFR > 25) at 6 months; **c** the absolute variation in the SRF of the operated kidney at scintigraphy (AV-SRF) at 6 months

The visual assessment of the association between WIT and functional endpoints univocally showed that no association was evident for values of WIT between 0 and 10 min, whereas above this limit a clear association could be detected (Fig. 1).

In detail, for WIT ≥ 10 min, the RCs were − 0.53 ml/min (95% CI − 0.99 to − 0.07, *p* = 0.024) and − 0.34 (95%CI − 0.63 to − 0.05, *p* = 0.022) for AV-GFR and AV-SRF, respectively, while the OR for RV-GFR > 25 was 1.13 (95%CI 1.03–1.25, *p* = 0.01). The differences in the median values of the endpoints between the patients that experienced a WIT < vs ≥ 10 min were: AV-GFR, − 3.7 (IQR − 12.1 to 1.6) vs − 7.5 (IQR − 18.3 to − 0.4) ml/min (p < 0.001); AV-SRF, − 1 (IQR − 5 to -1) vs -3.6 (IQR − 7.2 to − 1) % (*p* < 0.001); RV-GFR > 25, 9.3% vs 17.8% (*p* = 0.008).

Multivariable models confirmed the statistically significant associations reported above (Table 2). AV-GFR was found significantly associated to WIT ≥ 10 min (RC − 0.52, *p* = 0.019), age (RC − 0.35, *p* = 0.001) and baseline eGFR (RC − 0.30, *p* < 0.001), while AV-SRF showed a significant association with WIT ≥ 10 min (RC − 0.30, *p* = 0.018), baseline SRF (RC − 0.76, *p* < 0.001) and RENAL score (RC − 0.60. *p* = 0.028). WIT ≥ 10 min (OR 1.11, *p* = 0.007) and occurrence of AKI (19.7, *p* = 0.009) were significant predictors of RV-GFR > 25. The same analysis was performed excluding off-clamp patients and it confirmed the results of the overall cohort assessment (Supplementary Table 2).

**Table 2:** Multivariable models assessing predictors of relative variation (RV-GFR > 25%), absolute variation (AV-GFR) and absolute variation in the split renal function (AV-SRF)

	Odds ratio	95% Confidence interval	*p* value
**RV-GFR > 25**			
WIT < 10 min	0.98	0.88–1.10	0.854
WIT ≥ 10 min	1.11	1.02–1.20	**0.007**
Gender	1.03	0.50–2.16	0.918
Age (years)	1.03	0.99–1.07	0.069
Baseline eGFR	1.01	0.99–1.05	0.219
RENAL score	1.12	0.88–1.44	0.334
AKI	19.7	2.08–187.25	**0.009**

	Coefficient	95% Confidence interval	*p*-value
**AV-GFR**			
WIT < 10 min	0.004	– 0.40; 0.41	0.986
WIT ≥ 10 min	– 0.52	– 0.95; – 0.09	**0.019**
Gender	1.78	– 1.59; 5.15	0.299
Age (years)	– 0.35	– 0.55; – 0.15	**0.001**
Baseline eGFR	– 0.30	– 0.44; – 0.16	** < 0.001**
RENAL score	0.04	– 0.91; 1.07	0.94
AKI	– 10.02	– 27.41; 7.36	0.257
**AV-SRF**			
WIT < 10 min	0.062	– 0.17; 0.29	0.597
WIT ≥ 10 min	– 0.30	– 0.54; – 0.05	**0.018**
Gender	0.08	– 1.72; 1.88	0.931
Age (years)	– 0.02	– 0.08; 0.04	0.441
Baseline SRF	– 0.76	– 0.99; – 0.51	** < 0.001**
RENAL score	– 0.60	– 1.13; – 0.07	**0.028**
AKI	– 4.74	– 12.79; 3.31	0.247

*RV-GFR* > *25%* relative variation in eGFR over 25%, *AV-GFR* absolute variation of eGFR, *AV-SRF* absolute variation of split renal function at scintigraphy, *WIT* warm ischemia time, *eGFR* estimated glomerular filtration rate, *AKI* acute kidney injury

## Discussion

The present study analyzes a relatively consistent number of consecutive patients enrolled under the umbrella of a RCT. The study protocol imposed in all the enrolled patients to obtain a renal scintigraphy before and 6 months after surgery.

All surgical procedures respected some mandatory steps and were performed by a limited number of surgeons, featured by a homogeneous profile of experience. The main finding was that warm ischemia interval up to 10 min had no consequences on functional outcomes, while above this limit, there was a proportional and statistically significant functional impairment. However, these differences remained far from being clinically relevant, with negligible differences in global and split renal functions above and below 10 min of WIT (4 ml/min and 3%, respectively). Clearly, such considerations should be accepted only for patients with features similar those of patients enrolled by the CLOCK trial (two kidneys, regular baseline function, RENAL score ≤ 10).

In standard conditions, partial nephrectomy preserves 80% and 90% of ipsilateral and global eGFR, respectively, achieving a stable plateau in a few months after surgery [17, 18]. The nonmodifiable factors related to patient’s and tumor’s features play a dominant role on functional outcomes, as they reflect on the quality and quantity of residual nephrons [19]. Thus, the role of ischemia *per se* has been widely revised, even though remains substantial as the most readily factor modifiable through adaptations of surgical technique [20].

Theoretically, ischemia could injury nephrons with different mechanisms (vasoconstriction and abnormal endothelial response; tubular obstruction and urine backflow; reperfusion injury [21]) but the duration of ischemia after which an irreversible damage occurs still remains a matter of debate. Studies in CKD and single-kidney patients found a functional impairment proportional to warm ischemia that occurs early, after a few minutes [22].

Conversely, in patients with normal baseline renal function there is limited evidence on which is the optimal threshold of WIT. The majority of the authors accept a conventional limit of 25–35 min [23, 24]. Histopathological studies confuted such a threshold and showed that the human kidney can tolerate longer warm ischemia times [25, 26]. An additional challenge to deeply investigate postoperative function is the assessment of ipsilateral insult and SRF, accounting for contralateral compensation: only a few retrospective studies specifically investigated the functional results of RAPN by renal scintigraphy. These studies generally rely on small samples or subcohorts extracted from larger datasets where scintigraphy is not systematically indicated and therefore suffering from selection bias. Zargar and coll [27] reported on 99 RAPN—from a cohort of 666—with a median WIT of 20 min, showing that volume of healthy rim of renal parenchyma removed, WIT > 30 min, body mass index (BMI) and preoperative SRF were predictive of SRF preservation. Lee and coll [28] reported on 84 RAPN—from a cohort of 403 PN—with a mean WIT of 24 min, finding that baseline SRF and RENAL score were the significant predictors of postoperative SRF. Choi and coll [29] reported 229 RAPN with a mean WIT of 25 min, and found that tumor size, the “Nearness” (N) item of RENAL score, preoperative GFR, ischemia time and surgical approach predicted SRF. Luciani and coll [30] published a prospective study on 32 consecutive RAPN, with a median WIT of 24 min, investigated with an early scintigraphic control (1 month), showing that only tumor size predicted SRF. Matsura and coll [31] on 66 RAPN found that nephrometry, in detail the N item again, predicted SRF.

Altogether our results are in line with the above mentioned, but there are also some differences to be remarked. The median reduction in global eGFR in our cohort was − 5.5% (IQR − 15.9% to − 1%), and remained stable at further controls (data not presented: 12 months − 6.2%, 18 months − 5.3%, 24 months − 5.6%), confirming that RAPN leads to a mild global eGFR reduction stable along time. Such concept is in line with the findings by Porpiglia et al. who described renal damage status/postlaparoscopic PN stable over time within a cohort of 54 patients studied for a minimum follow-up of 4 years with radionuclide renal scan [23].

The median reduction in SRF was lower than previously reported (− 4.7%; IQR − 12.8% to 0%) and similar to the degree of the global reduction. This could be due to several factors: the timing of scintigraphic control, set at 6 months, i.e., out of the recovery phase for the ipsilateral kidney based on previous renal scan studies; differences in the methodology used to measure global function among studies; the inclusion of patients with regular baseline function, two normal kidneys and relatively small masses; the involvement of surgeons out of their learning curve probably reflecting in a minimal parenchymal sacrifice during resection and reconstruction. Similarly to other studies, we found that baseline eGFR and AKI impaired postoperative global renal function, and that baseline SRF and nephrometry were related to postoperative SRF. Finally, we demonstrated that WIT has a role on all functional outcomes according to a so-called “hinge threshold effect”: up to 10 min no significant differences were noted, while above there was a proportional functional impairment. Noteworthy, although statistically evident, the degree of impairment was almost clinically negligible, in agreement with our previous study referring to the primary endpoint of the trial [32]. Clearly, it cannot be denied that prolonging WIT an even clinically significant impairment will occur. As an example, the 6-months RV-GFR > 25% raised from 10% for WIT 0–10 min to 38.5% for WIT > 25 min (*p* < 0.001). However, in the entire cohort the median WIT was 10 min only, so that the low number of cases with longer times prevented more conclusive analysis. Definitely the CLOCK trial stated that ischemia during RAPN can be safely applied, when necessary [32], while the present study advises to adopt strategies to restrict WIT, when feasible. This is especially true for patients less resilient than those included in our trial, as with a single kidney or impaired baseline eGFR [23, 24].

This study, although coming from a prospective and controlled data collection regulated by the protocol of a RCT, is not devoid of limitations: first, the aim of this analysis was not the primary endpoint of the study; second, the amount of healthy tissue removed or damaged by surgery has not been objectively measured, but just approximate by nephrometry as surrogate; the inclusion criteria of the RCT restrict the validity of our conclusions to patients with similar features; we were not in the condition to test longer duration of WIT, given that the vast majority of cases remained under 16 mins.

## Conclusions

In the setting of bilateral kidneys, the application of warm ischemia for a time up to 10 min has no consequences on functional outcomes, while above this limit a proportional functional impairment was found, although not clinically relevant.

## Supplementary Information

Below is the link to the electronic supplementary material.

Supplementary table 1. Split cohort (clamp vs off-clamp) baseline, perioperative and pathological characteristics

Supplementary table 2. Multivariable models assessing predictors of relative variation (RV-GFR>25%), absolute variation (AV-GFR) and absolute variation in the split renal function (AV-SRF) excluding off-clamp cases

## Data Availability

Data will be available on request.
