# Supplementary material for: The role of warm ischemia time on functional outcomes after robotic partial nephrectomy: a radionuclide renal scan study from the clock randomized trial
Source: World J Urol. 2023 Apr 21;41(5):1337–44. doi: 10.1007/s00345-023-04366-3 (PMC10188582; doi:10.1007/s00345-023-04366-3)
Supplement: Supplementary file 1 — Supplementary table 1. Split cohort (clamp vs off-clamp) baseline, perioperative and pathological characteristics [file 345_2023_4366_MOESM1_ESM.docx]

|  |  |  |  |
| --- | --- | --- | --- |
|  | **Clamp** (206) | **Off-clamp** (118) | p-value |
| Age (years) | 64 (55-71) | 66 (56-70) | 0.76 |
| Gender (male) n (%) | 126 (61.2) | 69 (58.5) | 0.63 |
| BMI (Kg/m^2^) | 26 (24-28) | 27 (24-29) | 0.16 |
| Charlson Comorbidity Index | 0 (0-1) | 0 (0-1) | 0.63 |
| Hypertension n (%) | 115 (55.8) | 65 (55.1) | 0.81 |
| Diabetes n (%) | 22 (10.7) | 16 (13.6) | 0.13 |
| Vasculopathy n (%) | 36 (17.5) | 17 (14.4) | 0.77 |
| Cardiopathy n (%) | 50/205 (24.4) | 19/117 (16.2) | 0.59 |
| Pre-operative Hb (g/dL) | 14 (13-15) | 14 (13-15) | 0.47 |
| Creatinine at recruitment (mg/dL) | 0.83 (0.76-0.95) | 0.80 (0.67-0.96) | **0.04** |
| eGFR at recruitment (mL/min) | 86 (74-96) | 89 (81-99) | 0.15 |
| Split renal function operated kidney (%) | 48 (46-52) | 49 (47-51) | 0.27 |
| RENAL | 7 (6-8) | 6 (4-7) | **<0.001** |
| Tumor dimension (cm) | 3.3 (2.5-4.1) | 2.3 (2-3) | **<0.001** |
| EBL (mL) | 100 (50-150) | 100 (50-150) | 0.24 |
| WIT (minutes) | 15 (11-18) | - |  |
| OT (minutes) | 125 (107-160) | 115 (89-135) | **<0.001** |
| LOS (days) | 4 (3-5) | 4 (3-4) | **0.003** |
| Post-operative complications n (%) | 30 (14.6) | 16 (13.6) | 0.67 |
| Clavien grade n (%) |  |  | 1 |
| 1 | 20/29 (37.9) | 9 (56.2) |  |
| 2 | 11/29 (37.9) | 4 (25) |  |
| 3a | 5/29 (17.2) | 2 (12.5) |  |
| 3b | 2/29 (6.9) | - |  |
| 4a | - | 1 (6.2) |  |
| pT n (%) |  |  | 0.8 |
| 1a | 137/202 (67.8) | 97/112 (86.6) |  |
| 1b | 65/202 (32.2) | 15/112 (13.4) |  |
| Malignant histology n (%) | 157/201 (78.1) | 84/114 (73.7) | 0.39 |
| BMI=body mass index; eGFR=estimated glomerular filtration rate; EBL=estimated blood loss; WIT=warm ischemia time; LOS=length of stay | | | |
