# Supplementary material for: The role of warm ischemia time on functional outcomes after robotic partial nephrectomy: a radionuclide renal scan study from the clock randomized trial
Source: World J Urol. 2023 Apr 21;41(5):1337–44. doi: 10.1007/s00345-023-04366-3 (PMC10188582; doi:10.1007/s00345-023-04366-3)
Supplement: Supplementary file 2 — Supplementary table 2. Multivariable models assessing predictors of relative variation (RV-GFR>25%), absolute variation (AV-GFR) and absolute variation in the split renal function (AV-SRF) excluding off-clamp cases [file 345_2023_4366_MOESM2_ESM.docx]

| **RV-GFR>25** |  | | |
| --- | --- | --- | --- |
|  | *Odds ratio* | *95% Confidence interval* | *p-value* |
| **WIT<10 min** | 1.25 | 0.54-2.88 | 0.603 |
| **WIT≥10 min** | 1.12 | 1.03-1.21 | **0.004** |
| **Gender** | 1.45 | 0.60-3.49 | 0.404 |
| **Age (years)** | 1.03 | 0.99-1.08 | 0.113 |
| **Baseline eGFR** | 1.01 | 0.98-1.05 | 0.375 |
| **RENAL score** | 0.99 | 0.76-1.31 | 0.976 |
| **AKI** | 4.78 | 0.54-42.07 | 0.158 |
|  |  |  |  |
| **AV-GFR** |  |  |  |
|  | *Coefficient* | *95% Confidence interval* | *p-value* |
| **WIT<10 min** | 0.94 | -3.16; 1.28 | 0.404 |
| **WIT≥10 min** | -0.54 | -0.98; -0.11 | **0.015** |
| **Gender** | 1.53 | -2.58; 5.65 | 0.464 |
| **Age (years)** | -0.42 | -0.67; -0.18 | **0.001** |
| **Baseline eGFR** | -0.35 | -0.54; -0.16 | **<0.001** |
| **RENAL score** | 0.59 | -0.69; 1.89 | 0.364 |
| **AKI** | -0.42 | -28.00; 27.16 | 0.976 |
|  |  |  |  |
| **AV-SRF** |  |  |  |
|  | *Coefficient* | *95% Confidence interval* | *p-value* |
| **WIT<10 min** | -0.72 | -2.38; 0.94 | 0.393 |
| **WIT≥10 min** | -0.27 | -0.52; -0.03 | **0.029** |
| **Gender** | 1.00 | -1.35; 3.35 | 0.402 |
| **Age (years)** | -0.08 | -0.15; 0.0007 | 0.052 |
| **Baseline SRF** | -0.74 | -1.08; -0.41 | **<0.001** |
| **RENAL score** | -0.41 | -1.09; 0.28 | 0.24 |
| **AKI** | -7.25 | -19.42; 4.92 | 0.24 |
| *RV-GFR>25%= relative variation in eGFR over 25%; AV-GFR=absolute variation of eGFR; AV-SRF=absolute variation of split renal function at scintigraphy*  *WIT=warm ischemia time; eGFR=estimated glomerular filtration rate; AKI=acute kidney injury* | | | |
|  |  |  |  |
